# OnabotulinumtoxinA Add-On to Monoclonal Anti-CGRP Antibodies in Treatment-Refractory Chronic Migraine

**DOI:** 10.3390/toxins14120847

**Published:** 2022-12-02

**Authors:** Andreas A. Argyriou, Emmanouil V. Dermitzakis, Georgia Xiromerisiou, Michail Vikelis

**Affiliations:** 1 Headache Outpatient Clinic, Neurology Department, Agios Andreas State General Hospital of Patras, 26352 Patras, Greece; 2 Euromedica General Clinic, 54645 Thessaloniki, Greece; 3 Department of Neurology, Faculty of Medicine, School of Health Sciences, University Hospital of Larissa, 41110 Larissa, Greece; 4 Headache Clinic, Mediterraneo Hospital, 16675 Glyfada, Greece; 5 Glyfada Headache Clinic, 16675 Glyfada, Greece

**Keywords:** chronic migraine, treatment-refractory migraine, onabotulinumtoxinA, anti-CGRP monoclonal antibodies, dual therapy

## Abstract

We sought to assess the effectiveness of combining dual therapy with onabotulinumtoxinA (BTX) add-on to anti-calcitonin gene-related peptide (CGRP) monoclonal antibodies (anti-CGRP MAbs) in treatment-refractory patients with chronic migraine (CM). We retrospectively reviewed the medical files of 19 treatment-refractory patients with CM who had failed to two oral migraine preventatives, at least three consecutive BTX cycles (less than 30% response rate), at least three consecutive sessions with either fremanezumab or erenumab (less than 30% response rate), and were eventually switched to dual therapy with BTX add-on to any of the already-given anti-CGRP MAbs. We then assessed from baseline to each monotherapy or dual intervention predefined efficacy follow-up the changes in the following efficacy outcomes: (i) monthly headache days (MHD), (ii) monthly days with moderate/severe peak headache intensity, and (iii) monthly days with intake of any acute headache medication. Response (50% reduction in MHD) rates, safety, and tolerability were also determined. In the majority of cases (*n* = 14), dual targeting proved effective and was associated with clinically meaningful improvement in all efficacy variables; 50% response rates (also disability and QOL outcomes) coupled with favorable safety/tolerability. Our results advocate in favor of the view that dual therapy is effective and should be considered in difficult-to-treat CM patients who have failed all available monotherapies.

## 1. Introduction

Chronic migraine (CM) sufferers are those having headaches on at least 15 days per month (8 of these with typical migrainous features) for more than 3 months, with or without medication overuse headache (MOH) [1]. CM, accounting for less than 10% of diagnosed migraineurs and about 2% of the general population [2], is a much more debilitating condition than episodic migraine (EM), because CM patients experience aberrantly increased disability rates as a result of higher pain intensity, pain duration, pain-related comorbidities, pain-associated autonomic symptoms, and increased rates of MOH compared to EM sufferers [3]. In these CM patients, individualized acute and preventive therapeutic decisions should be made based on specific clinical phenotypes, previous pharmacological history and comorbidities, in order to reduce the frequency, severity, and duration of headache and likely achieve in converting CM to EM, thoroughly reducing disability, and improving health-related outcomes [4].

OnabotulinumtoxinA (BTX) was approved as a preventive treatment for CM in adults based on the outcomes of a pooled analysis of PREEMPT trials, which demonstrated a favorable benefit–risk ratio [5]. Clinically meaningful preventive antimigraine effects are exerted based on the ability of BTX to block vasoactive peptides, such as glutamate, calcitonin gene-related peptide (CGRP) and substance P from sensory nerve terminals, so as to directly suppress peripheral sensitization and neurogenic inflammation, coupled with indirect inhibition of central sensitization [6]. Several real-world studies confirm this excellent efficacy/safety BTX profile in CM settings [7]. On a national level, BTX has been approved to be fully reimbursed by the Greek National Health System as a third-line preventive treatment option in CM patients failing to respond to or who have not tolerated two previous first-line oral preventives.

The recent market release of anti-CGRP MAbs in Europe, based on the results of clinical trials and real-life data, appeared as a quite promising preventive option in both EM and CM patients to likely have a much higher efficacy–tolerability ratio compared to the already-available oral (in both EM and CM) and injectable prophylactic treatment armamentarium (BTX is only approved in CM). In Greece, fremanezumab was approved for reimbursement first and erenumab followed. However, considering cost-effectiveness, several European headache societies [8,9], recommend the use of anti-CGRP MAbs for CM patients having failed to respond to or who have not tolerated three previous prophylactic medications: two oral and necessarily BTX (after first delivering three consecutive courses per trimester). Hence, anti-CGRPs are currently fully reimbursed by the Greek National Health System (NHS) and social services, in line with the latter recommendation.

Nonetheless, not all CM patients adequately respond to injectable preventive therapies after commencing treatment with either BTX (at least three courses are required to be certain of non-responsiveness) or anti-CGRP MAbs (3–6 months’ treatment is warranted). By definition, treatment-refractory CM refers to the failure to bring the disease under acceptable control in response to all approved prophylactic therapies, either oral or injectable, with evidence of at least eight debilitating headache days per month for at least six consecutive months, while resistant migraine is defined by having failed at least three lines of migraine preventives and still experience at least eight debilitating headache days per month for at least three consecutive months without remission [10]. As such, according to the latter consensus statement of the European Headache Federation, patients with refractory CM are those most difficult to treat, pointing to the complexity of migraine pathophysiology to involve multiple pathways, mainly including modulation of sensory input in the trigeminovascular system, release from trigeminovascular neurons of vasoactive peptides, such as CGRP, glutamate and substance P to eventually evoke vasodilation and also neurogenic inflammation [11].

In such difficult-to-treat CM patients who fail to remit to available preventive treatments given as monotherapy, it was suggested by the recently updated American Headache Association consensus statement that dual targeting with BTX plus anti-CGRP Mab is probably effective and without significant safety signals [12], and thus deserving to be tested in selected individuals with sustained migraine and disability, based on direct or indirect effects of these preventives to block CGRP, and eventually evoke synergistic/additive antimigraine effects [13]. Thus far, the effectiveness of dual therapy with adding BTX to anti-CGRP MAbs has scarcely been addressed and the literature contains nine peer-reviewed publications [14,15,16,17,18,19,20,21,22], while there are some more data from abstract proceedings in international congresses [23]. Nonetheless, a recently published pooled analysis of real-world evidence supports anti-CGRP MAbs and BTX combined in CM [24]. Still, reimbursement of the latter dual therapy is not granted by the Greek NHS and patients with intractable CM wishing to try dual treatment have to be prescribed anti-CGRPs and to fully cover the cost of BTX.

We herein provide some additional data by reporting the outcome of a retrospective, multicenter study that sought to assess the effectiveness of combining dual therapy with BTX add-on to anti-CGRP MAb in treatment-refractory patients with CM who failed to respond (less than 30% response rate) on adequate monotherapy with three courses per trimester of BTX and subsequently with at least three monthly courses of either erenumab or fremanezumab.

## 2. Results

We reviewed the medical files of 19 consecutive treatment-refractory patients with CM who received at least two courses of BTX (per trimester) add-on to any anti-CGRP Mab (erenumab and fremanezumab per 28–30 days) after both of these interventions given as monotherapy had failed to provide at least a 30% response rate (reduction in monthly headache days) compared to baseline. The study sample consisted of 3 males (15.8%) and 16 females (84.2%) with a mean age of 42.1 ± 10.1 (range: 24–65) years, who attained combined treatment with BTX plus anti-CGRP MAb for a median time of 6 months (range 6–9 months). Table 1 describes in detail the baseline epidemiological and clinical characteristics of participants.

### 2.1. Changes in Efficacy Headache Outcomes after Dual Therapy Compared to Baseline and Each Monotherapy Intervention

**Figure 1:**
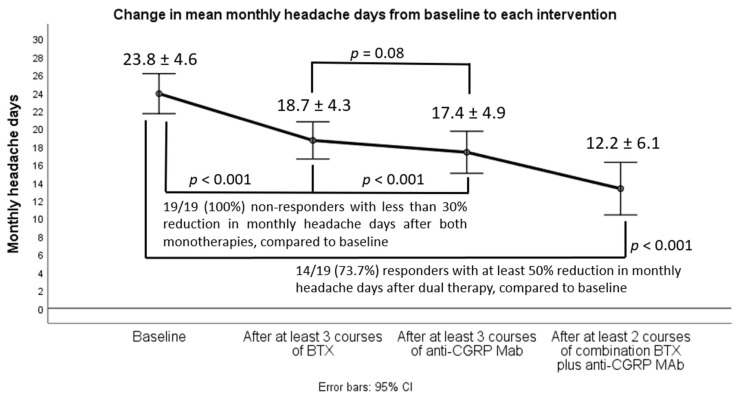
Changes in mean monthly headache days between baseline, monotherapy interventions and combined treatment.

**Figure 2:**
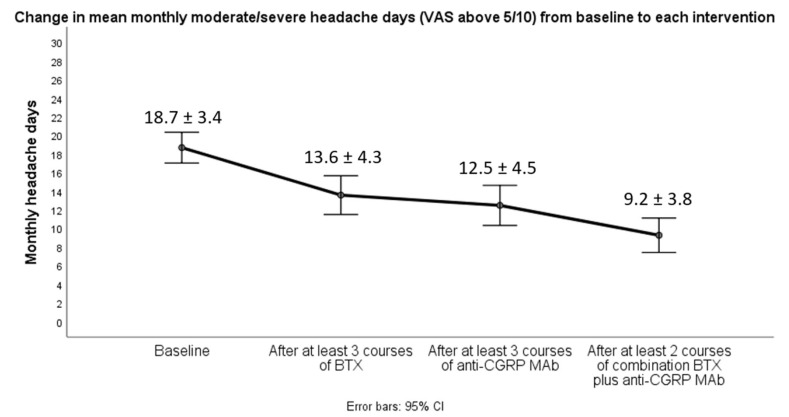
Changes in mean monthly days with peak headache intensity of at least 5 on the pain visual analogue scale between baseline, monotherapy interventions and combined treatment.

**Figure 3:**
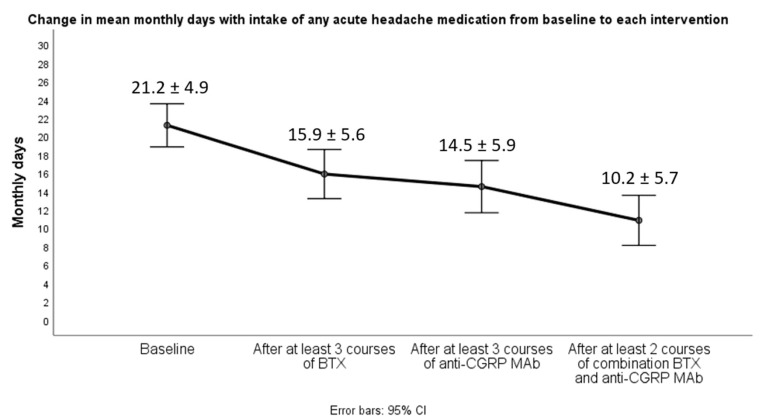
Changes in mean monthly days with intake of any acute migraine medication between baseline, monotherapy interventions and combined treatment.

A significant decrease (*p* < 0.001) in all efficacy variables was observed after at least two courses of the combined therapy compared either to baseline or at discontinuation of each monotherapy interventions. Notably, there was a statistically significant decrease in all efficacy variables after both monotherapy approaches compared to baseline, but this change was not clinically translated because all 19 patients did not achieve obtaining at least a 30% reduction in mean monthly headache days (MHD), being defined as non-responders at each monotherapy intervention. Switching from BTX to anti-CGRP MAb monotherapy (erenumab or fremanezumab) yielded no significant change (*p* = 0.08) in mean MHD (Figure 1). However, a majority of participants (14/19) only achieved converting from CM to EM (fewer than 15 MHD) and clinically meaningful de-escalation also in terms of headache attack severity and acute medication intake after initiation of dual therapy with BTX add-on to any of the already-given anti-CGRP MAbs (Figure 1, Figure 2 and Figure 3). In particular, CM patients with evidence of neck pain and tightness (*n* = 8) were those who mostly benefited from the dual intervention.

As mentioned, all participants were classified as non-responders after each monotherapy intervention, while 14 (73.7%) of them were eventually classified as responders after combined treatment, because they achieved response at 50% or more.

Just five (26.3%) patients remained strongly treatment-refractory (less than 50% reduction in MHD) to all approaches, either given as monotherapy or in combination.

### 2.2. Changes in Disability, Quality of Life and Satisfaction Scales after Dual Therapy Compared to Baseline and Each Monotherapy Intervention

**Figure 4:**
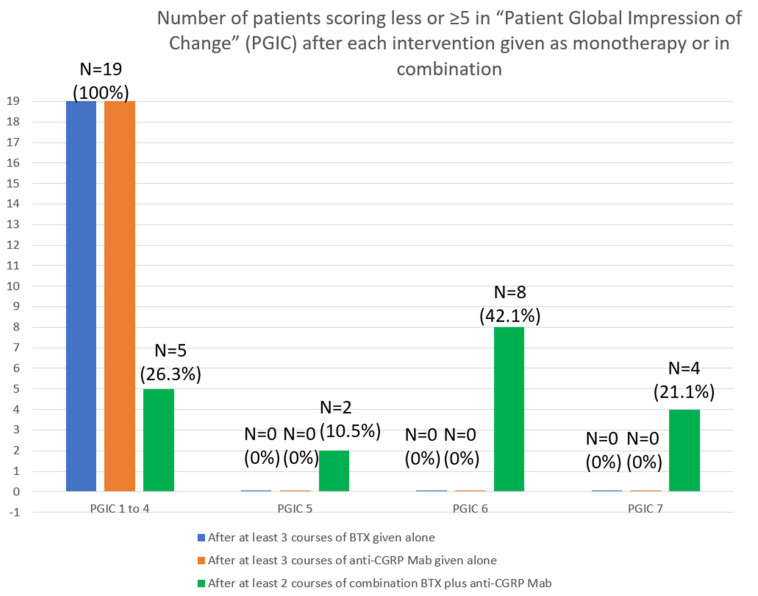
Differences in the number of patients scoring ≥ 5 in Patient Global Impression of Change scale (PGIC) after both monotherapy interventions and dual therapy.

The significant improvement, which was evident in efficacy headache outcomes solely after dual targeting, had an obvious impact on disability scale scores and was strongly associated with a significant reduction in MIDAS (138.4 ± 69.5 vs. 9.3 ± 4.8; *p* < 0.001) and HIT-6 (72.9 ± 4.6 vs. 60.6 ± 8.5; *p* < 0.001) scoring. The efficacy to dual therapy obviously influenced the perception of change and satisfaction of responders, as all of them (15/19) remained satisfied (score ≥ 5) on the Patient Global Impression of Change (PGIC) scale. Specifically, 2 scored 5, 8 scored 6, and 4 scored 7 (Figure 4).

Finally, the quality of life of responders to combined therapy, rated by EQ5D and EQVAS, was significantly improved to further document the merits of delivering dual direct and indirect CGRP targeting in treatment-refractory CM patients.

### 2.3. Safety Analysis

Concerning the safety/tolerability profile of dual therapy, there was no single adverse event (AE) or serious adverse event (SAE) experienced by ≥5 patients as we only registered mainly mild and transient side effects at rates, in keeping with the known safety profile of both BTX and anti-CGRPs to include pain, erythema or wheals in the injection site; mild ptosis and eyebrow elevation (the latter for BTX). No systemic adverse events were recorded as a result of anti-CGRP administration. There were no cases of early withdrawal from dual intervention due to safety/tolerability issues.

## 3. Discussion

The dual antimigraine preventive therapy with available injectables in CM is poorly investigated, as clearly documented in a recently published systematic review and pooled analysis, wherein only 8 out of 329 studies from initial screening met the inclusion criteria, of which just 5 were subjected to qualitative meta-analysis. A majority of analyzed studies were retrospective chart reviews and just one prospectively addressed the efficacy/safety of dual therapy [16]. The beneficial risk–benefit ratio of the combined treatment was clearly documented in all retrospective studies, as the dual approach with erenumab plus BTX was superior to monotherapy in terms of 50% reduction of MHD and monthly migraine days (MMD), while the mean MIDAS and HIT-6 scores were also significantly reduced. No significant safety signals were evident, apart from mild AEs, mainly including pain in the injection sites, skin changes, constipation and hair loss [14,15,17,19,20]. More robust evidence to support the beneficial effect of dual therapy was revealed in the prospective (the only available) observational study, which clearly showed that combining erenumab with BTX provided a significantly higher reduction in migraine frequency in 65% of patients compared to treatment with erenumab alone (26%) or with erenumab in combination with any prophylactic treatments (15%) other than BTX [16]. Fremanezumab yielded a similar beneficial risk–benefit ratio when it was combined with BTX [22]. Only one recently published study found comparable rates in 50% reduction in MHD and MMD, demonstrating as such non-superiority of dual treatment compared to monotherapy alone [18].

In the current setting, we sought to further test the efficacy–safety profile of dual therapy in a well-characterized cohort of treatment-refractory patients with CM who had failed to remit after administration of all available preventives, either oral or injectable (less than 30% response rate) in a stepwise manner, as instructed by international and national guidelines. In agreement with available knowledge on the topic, we confirm that patients treated with the combination therapy, comprised of BTX add-on to anti-CGRP MAb, achieved a reduction in MHD, which was much higher than that obtained with either monotherapy alone. Likewise, 50% of responders were clearly superior in dual-targeted patients compared to monotherapies. Patients with evidence of neck pain and tenderness were those who benefited most from the dual intervention, apparently as a result of BTX rather than of the anti-CGRP Mab administration. No safety/tolerability issues emerged. Acknowledging methodological limitations, including the retrospective design and the small number of included patients, which might have prevented the results from being extrapolated, our results taken together thoroughly demonstrate a positive benefit–risk ratio to eventually further support the argument that dual antimigraine preventive therapy is effective and should be pursued in treatment-refractory CM patients in order to achieve treatment optimization. The national policy that is currently applied in Greece for reimbursing injectable antimigraine prophylactics restricted us from having a larger sample size, as many patients were reluctant to directly pay on their own expenses a significant amount of money for maintaining combination therapy (had to pay for two BTX 100 UI flacons in private pharmacies—about EUR380) in these difficult financial times. Nonetheless, we should note that the retrospective design we applied is similar to that followed in most relevant publications, thus potentially making the current results directly comparable to those previously reported.

Whether the efficacy of combining BTX and anti-CGRP MAb is attributed to additive (combined effect) rather than synergistic (sum of separate actions) effect remains a matter of debate. An additive effect is more likely to occur, as BTX and anti-CGRP MAb seem to have distinct mechanisms of action, the first by preventing the activation and sensitization of unmyelinated neuronal C-wide dynamic range (C-WDR) afferents without affecting the thinly myelinated Aδ-high threshold (Aδ-HT) fiber nociceptors [25]. On the contrary, anti-CGRP MAbs selectively inhibit sustained firing of Aδ-HT fibers in the trigeminal ganglion, but not C-WDR afferents [26,27]. This is particularly evident for fremanezumab (our sample was mostly fremanezumab-treated; 17/19 participants), as this was thoroughly experimentally demonstrated in rats [26]. However, one could argue that the selective targeting of these different types of nociceptive fibers is peculiar, because both pathways transmitting pain signals seem closely interrelated [13]. In support of the latter view, there is evidence to demonstrate that firing of Aδ-HΤ fibers remains dependent on CGRP release of C-WDR nociceptors in synaptic terminals, as also in the axo-axonic synapses along the nodes of Ranvier of the afferent pathways [28]. Furthermore, inhibition of CGRP by anti-CGRP MAbs is able to evoke direct blocking of Aδ-HΤ pathways, whereas both direct C-WDR fiber inhibition and indirect Aδ-HT pathway inhibition occurs with BTX, because CGRP release is blocked from C fibers [13]. Hence, one cannot rule out with confidence that BTX can induce both direct inhibition of C-WDR nociceptors in the spinal trigeminal nucleus and an indirect inhibition of Aδ-HT fibers in the dura. On the contrary, the inhibitory effect of anti-CGRP MAbs selectively upon Aδ-HT fibers is robustly established [26], while good response to anti-CGRP MAbs can be predicted, among others, by evidence of decreased baseline mean flow velocity in middle cerebral arteries [29].

## 4. Conclusions

To conclude, we were hopefully able to further demonstrate in our real-world setting that adding BTX to anti-CGRP MAb in treatment-refractory patients, with CM being associated with clinically meaningful improvement in all efficacy variables, 50% response rates (also disability and QOL outcomes), and favorable safety/tolerability. As such, with our results, we advocate in favor of the view that dual therapy is indeed effective and should be considered in difficult-to-treat CM patients who have failed to all available monotherapies, either orally or injectable administered, in order to likely increase the potential for additive/synergistic effects so as to eventually provide adequate pain relief and significant reduction in MHD. However, considering the small sample, further well-designed, sufficiently powered prospective studies are warranted to shed additional light on this clinically important issue concerning the adequate management of treatment-refractory CM.

## 5. Materials and Methods

After obtaining written informed consent from each patient, we conducted a multicenter, retrospective chart review, which was in line with the principles of the Helsinki Declaration. Adult (18–65 years) CM patients with or without MOH, according to the 2018 criteria of the International Classification of Headaches Disorders-III [1], were included in this study if they (i) were considered non-responders to at least 2 preventive oral medications because of inefficacy or intolerance, (ii) switched to BTX according to the approved indications, standard clinical practice, and national reimbursement policies and remained nonresponsive (less than 30% reduction in MHD) after at least 3 consecutive courses per trimester of 155–195 UI of BTX, (iii) had again switched according to the approved indications and national reimbursement policies to any market-available anti-CGRP Mab in Greece, namely, erenumab or fremanezumab, and still failed to achieve a 30% response rate after receipt of at least 3 monthly consecutive courses, (iv) were eventually continued on prescribed erenumab or fremanezumab (fully reimbursed) and agreed to individually cover the economic cost of 2 BTX 100 UI flacons (self-paid) in order to receive dual preventive therapy with BTX 155–195UI commenced every 3 months as add-on to erenumab 140 mg or fremanezumab 225 mg injected monthly, (v) had agreed to keep headache diaries and fill in migraine disability scales as instructed (headache diary compliance was set to at least 80% of total days), and (vi) were consistent in providing safety/tolerability data through monthly phone interviews. We excluded patients having been previously exposed before study entry to any injectable migraine preventive treatment and those with presence of major psychiatric disorder. Pregnant or nursing females were also excluded.

BTX (Botox^®^ 100UI/fl; Allergan-Abbvie, Hellas, Greece) either as monotherapy or as part of the dual intervention was administered from certified BTX injectors at predefined cranial and cervical sites and at a fixed dose of 155–195 UI per trimester, according to our previously published experience up to 5 years of continuous (per trimester) BTX exposure [30,31,32,33]. Patients on BTX monotherapy were scheduled to receive at least three treatment cycles per trimester before defining success or failure to intervention. BTX responders were those with at least 50% reduction in MHD after being treated with 3 consecutive BTX courses.

Erenumab 140 mg every 28 days (Aimovig^®^; Novartis Pharma, Athens, Greece) or fremanezumab 225 mg every 30 days (Ajovy^®^; TEVA Pharma, Athens, Greece) were administered according to standard clinical practice for at least 3 courses. Same criteria of clinical response were maintained (50% response rate) to assess the efficacy of anti-CGRP MAbs either given as monotherapy or combined with BTX.

Patients’ headache diaries were retrieved from their medical files, kept in the corresponding headache outpatient clinics, and used in order to serve our primary objective, which was to document potential differences in clinical headache outcomes after each monotherapy or dual treatment compared to baseline or between interventions. Headache efficacy variables during the whole exploratory treatment period included (i) the crude efficacy of monotherapies or dual therapy as expressed by the change in mean number of MHD compared to baseline and comparatively (BTX alone vs. anti-CGRP alone) between monotherapies, (ii) change in mean MHD with peak moderate/severe intensity (more than 4 out of 10 on a 0–10 numerical scale) of cephalalgic pain from baseline to each (and between) single or dual therapy, and (iii) change in days with intake of any acute headache medications, either triptans, nonsteroidal anti-inflammatory drugs, or other, from baseline to each intervention. We generally considered patients with at least 50% reduction in MHD after each intervention compared to baseline as responders, while the number of responders between monotherapies and dual targeting was compared to document the corresponding degree of efficacy in this sample of quite difficult-to-treat CM patients.

Secondary objectives included the documentation of effects from each single or dual approach on scores of migraine disability scales, i.e., Migraine Disability Assessment questionnaire (MIDAS) [34], Headache Impact Test-6 (HIT-6) [35] and EQ5D, which is a questionnaire used to capture the perceived changes in health outcomes and quality of life (QOL) of patients after the initiation of treatments. EQ-5D is comprised of the “self-classifier” part to which participants are asked to self-rate on a 3-point scale (1 stands for no problems and 3 for extreme problems), the “five dimensions of health status,” i.e., mobility, self-care, usual activity, pain/discomfort, anxiety/depression, and the second part is EQ-VAS, in which the responders are instructed to again self-rate their health state on a thermometer vertical scale ranging from 0–100 with scores from 0–40 to suggest severe impairment of health state due to pain or psychological distress. Scores of 50–70 to underlie moderate affectation of health state, and scores of 80–100 point at excellent health state [36]. Finally, the short self-report 7-point (1 stands for “no change” and 7 for “considerable improvement”) patients’ reported outcome (PRO) questionnaire Patient Global Impression of Change (PGIC) was used to rate the patients’ self-perceived impact of disease management and satisfaction from baseline to the predefined efficacy follow-up each single or combined intervention [37]. Patients with PGIC ≥ 5 were defined those with “clinically significant benefit,” while a PGIC ≤ 4 scoring was linked to “no benefit,” according to the IMMPACT recommendations [38]. Safety and tolerability evaluation was performed by phone contacts with patients at day 21 following every BTX or anti-CGRP Mab infusion and any local or systemic AE was recorded in their medical files.

### Statistical Analysis

Descriptive statistics were computed depending on the nature of the analyzed variable. The changes in subsequent clinical efficacy scores from baseline to each monotherapy or dual approach were first assessed using paired-sample *t*-tests, after checking the normal distribution of the variables with the Kolmogorov–Smirnov test. Moreover, the consistency of the longitudinal changes documented at the predefined subsequent efficacy evaluation follow-ups was tested with repeated-measures ANOVA with Bonferroni correction. The same approach was followed to assess secondary outcomes. Unless otherwise stated, all tests were two-sided, and significance was set at *p* < 0.05. SPSS for Windows (release 27.0, SPSS Inc., Chicago, IL, USA) calculated the statistics.

## Figures and Tables

**Table 1:** Demographic and clinical characteristics of participants at baseline.

Participants n = 19	**N (%)**
***Variable***	
**Gender**	
Females	16 (84.2)
Males	3 (15.8)
**Age ± SD (range)**	42.1 ± 10.1 (24–65)
**Previous lines of prophylactic medications** Median value (range)	5 (3–7)
**Years ± SD (range) with chronic migraine**	10.1 ± 3.9 (6–18)
**Psychiatric comorbidities**	17 (89.5)
Anxiety disorder	3
Depression	4
Mixed anxiety and depression disorder	9
Bipolar disorder	1
**Medication overuse headache**	
Yes	17 (89.5)
No	2 (10.5)
**Previous courses of BTX** median (range)	4 (3–5)
**Previous courses of anti-CGRP MAb** median (range)	5 (3–7)
**Specific anti-CGRP Mab**	
Erenumab 140 mg every 28 days	2 (10.5)
Fremanezumab 225 mg every 30 days	17 (89.5)

## Data Availability

The data that support the findings of this study are available from the corresponding author upon reasonable request.
